# Pharmacological Inhibition and Genetic Deletion of Cystathionine Gamma-Lyase in Mice Protects against Organ Injury in Sepsis: A Key Role of Adhesion Molecules on Endothelial Cells

**DOI:** 10.3390/ijms241713650

**Published:** 2023-09-04

**Authors:** Sumeet Manandhar, Stephen Chambers, Andrew Miller, Isao Ishii, Madhav Bhatia

**Affiliations:** 1 Department of Pathology and Biomedical Science, University of Otago, Christchurch 8140, New Zealand; sumeet.manandhar@postgrad.otago.ac.nz (S.M.); steve.chambers@otago.ac.nz (S.C.); andrew.miller@otago.ac.nz (A.M.); 2 Department of Health Chemistry, Showa Pharmaceutical University, Machida, Tokyo 194-8543, Japan; i-ishii@ac.shoyaku.ac.jp

**Keywords:** hydrogen sulfide, cystathionine gamma-lyase, sepsis, adhesion molecules, endothelial cells

## Abstract

Hydrogen sulfide (H_2_S), synthesized by cystathionine gamma-lyase (Cth), contributes to the inflammatory response observed in sepsis. This study examines the effect of Cth-derived H_2_S in adhesion molecules on endothelial cells of vital organs in mice in a cecal ligation puncture (CLP)-induced model of sepsis, using two different and complementary approaches: Cth gene deletion and pharmacological inhibition. Our findings revealed a decreased level of H_2_S-synthesizing activity (via Cth) in both Cth^−/−^ mice and PAG-treated wild-type (WT) mice following CLP-induced sepsis. Both treatment groups had reduced MPO activity and expression of chemokines (MCP-1 and MIP-2α), adhesion molecules (ICAM-1 and VCAM-1), ERK1/2 phosphorylation, and NF-κB in the liver and lung compared with in CLP-WT mice. Additionally, we found that PAG treatment in Cth^−/−^ mice had no additional effect on the expression of ERK1/2 phosphorylation, NF-κB, or the production of chemokines and adhesion molecules in the liver and lung compared to Cth^−/−^ mice following CLP-induced sepsis. The WT group with sepsis had an increased immunoreactivity of adhesion molecules on endothelial cells in the liver and lung than the WT sham-operated control. The Cth^−/−^, PAG-treated WT, and Cth^−/−^ groups of mice showed decreased immunoreactivity of adhesion molecules on endothelial cells in the liver and lung following sepsis. Inhibition of H_2_S production via both approaches reduced adhesion molecule expression on endothelial cells and reduced liver and lung injury in mice with sepsis. In conclusion, this study demonstrates that H_2_S has an important role in the pathogenesis of sepsis and validates PAG use as a suited tool for investigating the Cth/H_2_S-signalling axis in sepsis.

## 1. Introduction

Sepsis is a major public health problem with an overall mortality rate of 25–30% globally [1]. The most common cause is bacterial infection [2], which may trigger a systemic inflammatory response and multiple organ failure [3]. Severe liver injury and lung injury may be associated with sepsis. The underlying mechanisms of injury are not well understood but recent studies have shown H_2_S is an important gaseous mediator of inflammation [4,5,6,7]. 

H_2_S is synthesized through the metabolism of l-cysteine, in coordination with PLP (pyridoxal 5′-phosphate), via three enzymes: cystathionine γ-lyase (Cth), cystathionine β-synthase (CBS), and 3-mercaptopyruvate sulfurtransferase (MPST) [8]. Cth is predominantly responsible for H_2_S synthesis in the liver, whereas CBS mainly regulates H_2_S synthesis in the brain and kidneys [9]. H_2_S synthesized via Cth plays an essential role in physiological as well as in pathological processes [10]. It has been demonstrated that H_2_S acts as a pro-inflammatory mediator during various acute inflammatory conditions [7,11,12], including cecal ligation and puncture (CLP)-induced sepsis [13,14,15] in mice as well as in patients with sepsis [16,17]. CLP-induced sepsis increased Cth expression/H_2_S synthesis in these studies, promoting the inflammatory response by elevating pro-inflammatory cytokines, chemokines, and adhesion molecules [11,12,13]. These pro-inflammatory mediators were increased via the activation of NF-κB under the influence of Cth expression/H_2_S synthesis during the inflammatory episode [18]. In addition, Cth expression/H_2_S synthesis have been shown to regulate inflammation via the activation of extracellular signal-regulated kinase (ERK) [19], which is the primary upstream activator of NF-κB [18]. 

Recruitment of leukocytes is a key feature of inflammation. This involves a series of events that results in the recruitment of leukocytes to the site of inflammation via leukocyte–endothelial cell interactions [20]. This series of events is initiated by rolling and then adherence of leukocytes to the endothelial cells via the interaction between the adhesion molecules and their respective ligands. This, in turn, results in the infiltration of leukocytes to the site of injury [21,22]. Increased concentrations of H_2_S have been reported to significantly increase pulmonary and hepatic concentrations of chemokines and adhesion molecules during sepsis [18,23].

Liver sinusoidal endothelial cells (LSECs) are the most abundant non-parenchymal cells in the liver, with unique functions, especially in leukocyte recruitment via various adhesion molecules expressed on their surface [24,25]. LYVE-1 (lymphatic vessel endothelial hyaluronan receptor-1) has been used as a potential maker for liver sinusoidal endothelial cells. Adhesion molecules such as intercellular adhesion molecule 1 (ICAM-1) and vascular cell adhesion molecule 1 (VCAM-1) [22,26] are highly expressed on inflamed LSECs [27]. We have found, in previous studies, that sepsis is associated with the disruption of the LSECs and the formation of gaps [4,28,29], which are large defects through the LSECs. Cth gene deletion protects mice against disruption of the LSECs caused by sepsis, suggesting disruption of LSECs’ structure is an important consequence of H_2_S-induced inflammation in sepsis [4]. In parallel, acute lung injury (ALI), and its more severe form ARDS (acute respiratory distress syndrome), are manifestations of lung injury that occur during sepsis [30]. Pulmonary endothelial cells have been identified as key modulators and orchestrators of ALI via leukocyte infiltration [31]. Expression of ICAM-1 and VCAM-1 are reported to be increased in the pulmonary endothelial cells during ARDS, which can be associated with septic shock and multiple organ failure [32]. Many studies have employed CD-31 (PECAM-1) as a potential marker for pulmonary endothelial cells. Inhibition of Cth expression/H_2_S synthesis has been shown to protect against lung injury induced by sepsis [4,14,15,18,19]. To date, various methods have been used to understand the role of H_2_S in inflammation related to sepsis, such as pharmacological tools [14,15,18,19], siRNA, and knockout mouse models [4]. These tools have opened up a new horizon for gaining deeper insights into the role of H_2_S in inflammation. The most commonly used pharmacological tool is dl-propargylglycine (PAG). It is a specific inhibitor of Cth with no role in the inhibition of CBS. However, some studies have reported that PAG acts by inhibiting other PLP-dependent enzymes as well [33], leading to questions about its specificity.

Interestingly, a recent study with Cth deletion mice showed similar results to previous studies using PAG [14,15,18,19], indicating that the actions of PAG were indeed a result of Cth inhibition. The current study, therefore, uses two complementary and independent approaches to investigate the role of H_2_S during sepsis. Moreover, so far, no study has explored the role of the Cth/H_2_S signaling pathway on adhesion molecules expressed by the endothelial cells of vital organs during sepsis. Although previous studies have pointed to a key role of LSECs in the pro-inflammatory actions of H_2_S, the mechanism by which they contribute to inflammation in sepsis remains unknown. 

In this study, therefore, we aimed to investigate whether H_2_S, synthesized via Cth, plays a role in the alteration of adhesion molecule expression on endothelial cells of the vital organs in CLP-induced septic mice. To achieve this, we utilized Cth^−/−^ mice and PAG-treated wild-type Cth^−/−^ mice, and examined the impact of different approaches to Cth inhibition on liver and lung pro-inflammatory mediators, injury, and adhesion molecule alteration in the endothelial cells in a mouse model of sepsis. This parallel use of two complementary and independent means of H_2_S inhibition will help us to gain strong evidence of the role of endogenously synthesized H_2_S in inflammation.

## 2. Results

### 2.1. Cth Expression and H_2_S-Synthesizing Enzyme Activity

**Figure 1:**
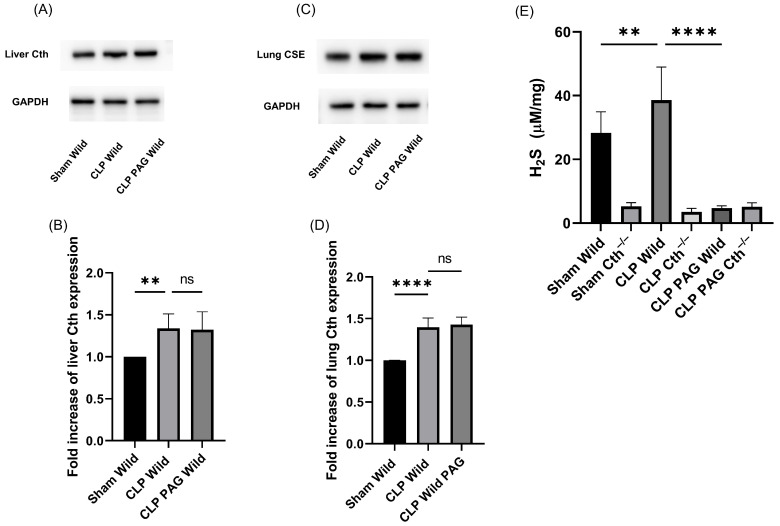
Cth expression in the liver (**A**,**B**) and lung (**C**,**D**) and liver H_2_S production (**E**) after 8 h of CLP-induced sepsis. The expression of Cth protein in both liver and lungs was induced by CLP-induced sepsis. Cth band density was normalized to GAPDH band density. Results are expressed in relative fold increase. Representative Western blot images (**A**,**C**) were obtained from at least four independent experiments. (**E**) Liver H_2_S-synthesizing activity measured over 30 min. In WT CLP-induced sepsis mice, H_2_S-synthesizing activity was higher than in sham controls, whereas significantly lower concentrations were observed in Cth^−/−^ mice. CLP-induced sepsis in WT and Cth^−/−^ treated with demonstrated similar concentrations of H_2_S-synthesizing activity to sham Cth^−/−^. All data are represented as mean ± standard derivation (SD) (*n* = 8). One-way ANOVA with post hoc Tukey test was employed. Statistical significance was determined at ** *p* < 0.01; **** *p* < 0.0001; ns, not significant.

The expression of Cth protein in both the liver and lung was significantly higher in WT CLP-induced septic mice than in sham control mice (Figure 1A–D). However, treatment with the Cth pharmacological inhibitor PAG did not affect the expression of Cth protein in WT CLP-induced septic mice.

The liver H_2_S-synthesizing enzyme activity in WT mice was significantly higher following CLP-induced sepsis than in the sham-operated control. It was significantly lower in Cth^−/−^ mice than WT CLP-induced sepsis, and CLP-induced sepsis did not increase H_2_S-synthesizing enzyme activity in Cth^−/−^ mice compared with sham Cth^−/−^. In addition, the treatment of PAG in both WT and Cth^−/−^ mice showed similar H_2_S-synthesizing activity in the liver. We could not detect any traces of H_2_S-synthesizing activity in homogenized lung samples from any experimental group (Figure 1E). 

### 2.2. Effect of PAG Treatment and Cth^−/−^ on Myeloperoxidase (MPO) Activity and Sepsis-Associated Organ Injury

**Figure 2:**
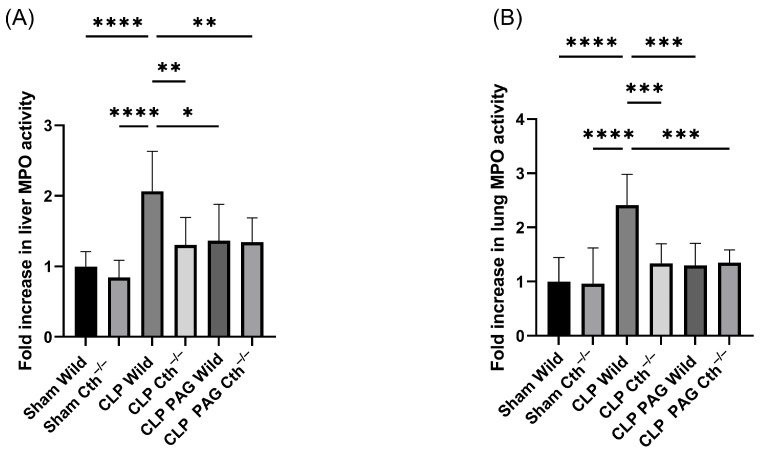

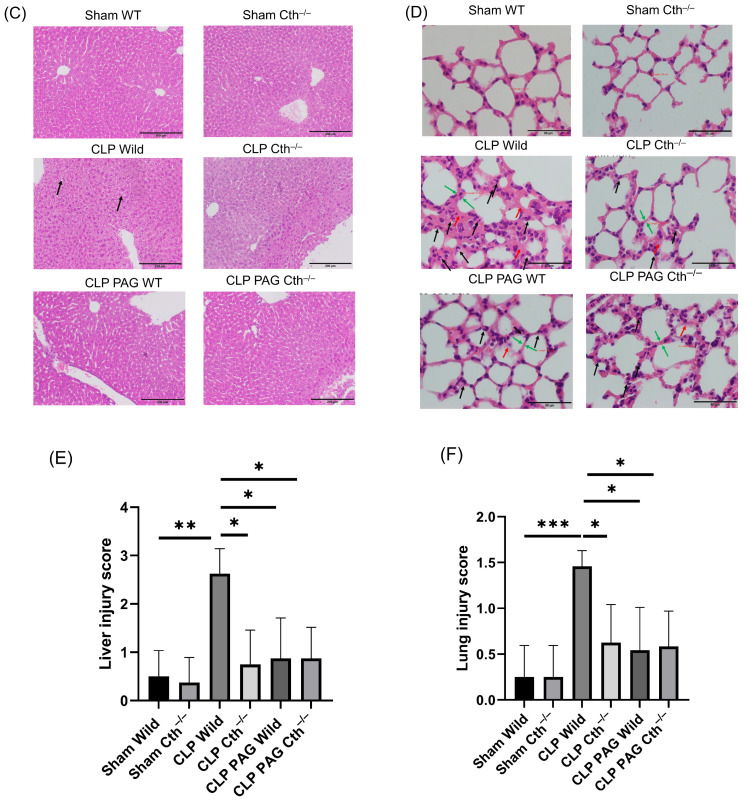
Impact of Cth inhibition or deletion on liver and lung injury after 8 hr of CLP-induced sepsis. MPO activity in the liver (**A**) and lung (**B**). Compared to sham operation controls, liver and lung MPO activity were higher in WT CLP-induced septic mice. CLP-induced sepsis in WT, Cth^−/−^, PAG-treated WT, and PAG-treated Cth^−/−^ significantly reduced MPO activity in the liver and lung. MPO activity was expressed as a relative fold increase over sham operations. (**C**) Representative histopathological images of the liver showed severe ballooning degeneration (liver injury) in WT CLP-induced sepsis mice (two large ballooned hepatocytes—black arrows) compared to sham control. CLP-induced Cth^−/−^, PAG-treated WT, and PAG-treated Cth^−/−^ mice revealed reduced level liver injury (less ballooning degeneration) (**E**); Scale bars = 200 μm. (**D**) Representative histopathological images of the lung. Histological analysis of lung sections showed prominent lung injury (fibrin deposition (red arrows), leukocyte (neutrophil) infiltration (black arrows), and alveolar thickening (green arrows)) (**F**) in WT mice following CLP-induced sepsis, compared to sham-operated controls. Notably, this effect was significantly diminished in Cth^−/−^, PAG-treated WT, and PAG-treated Cth^−/−^ mice following CLP-induced sepsis. Scale bars  =  50 μm. One-way ANOVA with post hoc Tukey test was employed for liver and lung MPO activity, whereas non-parametric Kruskal–Wallis test was used to analyze liver and lung histology scores. All data are represented as mean ± SD (*n* = 8), and statistical significance was determined at * *p* < 0.05; ** *p* < 0.01; *** *p* < 0.001; and **** *p* < 0.0001.

Liver and lung MPO activity were measured to assess neutrophil infiltration, and the increment in MPO activity over baseline was used as marker of neutrophil infiltration into these organs (Figure 2A,B). There was increased MPO activity in both the liver and lungs of mice with WT CLP-induced sepsis compared to sham control mice. PAG-treated WT and Cth^−/−^ mice following CLP had significantly less MPO activity in both the liver and lung than mice with WT CLP-induced sepsis. Histological analysis of liver tissue sample from WT mice with sepsis showed more severe liver injury (ballooning degeneration) compared to sham WT, sham Cth^−/−^, CLP Cth^−/−^, CLP PAG WT, and CLP PAG Cth^−/−^ (Figure 2C). Less lobular necrosis was observed in samples from mice in the CLP Cth^−/−^, CLP PAG WT, and CLP PAG Cth^−/−^ groups, but not in the sham WT and sham Cth^−/−^ groups compared to WT mice with sepsis. Similarly, lung sections stained with H&E showed more evidence of lung injury (fibrin deposition, leukocyte infiltration, and thickening of alveolar walls) in septic WT mice compared to septic mice with Cth^−/−^, PAG-treated WT, and Cth^−/−^. Normal lung histological structure was seen in WT sham-operated and Cth^−/−^ mice (Figure 2D,E).

### 2.3. Effect of PAG Treatment and Cth^−/−^ on Liver and Lung ERK 1/2/NF-κB in Sepsis

**Figure 3:**
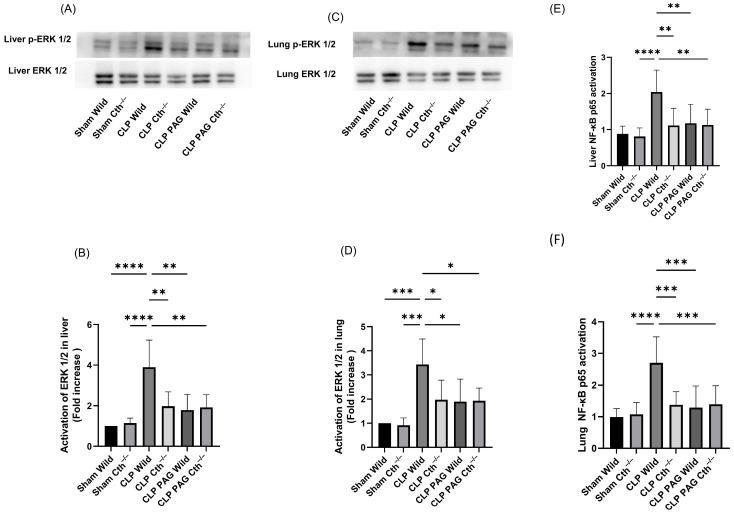
Impact of Cth inhibition or deletion on ERK1/2 and NF-κB activation after 8 h of CLP-induced sepsis. (**A**,**B**) p-ERK1/2 expression in the liver. WT CLP-induced septic mice showed increased phosphorylation of ERK1/2 as compared to the WT sham-operated mice. CLP-induced sepsis in Cth^−/−^, PAG-treated WT, and PAG-treated Cth^−/−^. ERK1/2 phosphorylation was significantly lower than WT CLP-induced sepsis. (**C**,**D**) p-ERK1/2 expression in the lung. WT CLP-induced septic mice showed increased phosphorylation of ERK1/2 compared to the WT sham-operated mice. CLP-induced sepsis in Cth^−/−^, PAG-treated WT, and PAG-treated Cth^−/−^. ERK1/2 phosphorylation was significantly lower than WT CLP-induced sepsis. p-ERK1/2 band density was normalized to ERK1/2 band density. Results are expressed in relative fold increase. Representative Western blot images were obtained from at least four independent experiments. (**E**,**F**) NF-κB p65 activation in the liver and lung. Activation of NF-κB p65 was higher in WT CLP-induced septic mice than WT sham-operated mice. CLP-induced sepsis in Cth^−/−^, PAG-treated WT, and PAG-treated Cth^−/−^ activation of NF-κB p65 was significantly lower than WT CLP-induced sepsis. The results are expressed as a relative fold increase. All data are represented as mean ± SD (*n* = 8). One-way ANOVA with post hoc Tukey test was employed. Statistical significance was determined at * *p* < 0.05; ** *p* < 0.01; *** *p* < 0.001; and **** *p* < 0.0001.

ERK phosphorylation was higher in the liver and lung samples from WT mice with CLP-induced sepsis than WT sham-operated mice. As expected, phosphorylation was lower in samples from both the PAG-treated and Cth^−/−^ with CLP-induced sepsis compared to the corresponding WT mice. No additional effect was observed with the treatment with PAG in Cth^−/−^ mice followed by CLP-induced sepsis compared with either group alone (Figure 3A–D). Nuclear NF-κB DNA-binding activity in the lungs and liver was significantly increased in WT CLP-induced septic mice compared to WT sham-operated mice. Blockade of endogenous H_2_S synthesis with PAG-treated or Cth^−/−^ mice showed a marked inhibition of activation of NF-κB, and this was unaffected by PAG treatment in Cth^−/−^ mice with sepsis (Figure 3E,F).

### 2.4. Effect of PAG Treatment and Cth^−/−^ on Pro-Inflammatory Chemokines Synthesis on Liver and Lung Following Sepsis

**Figure 4:**
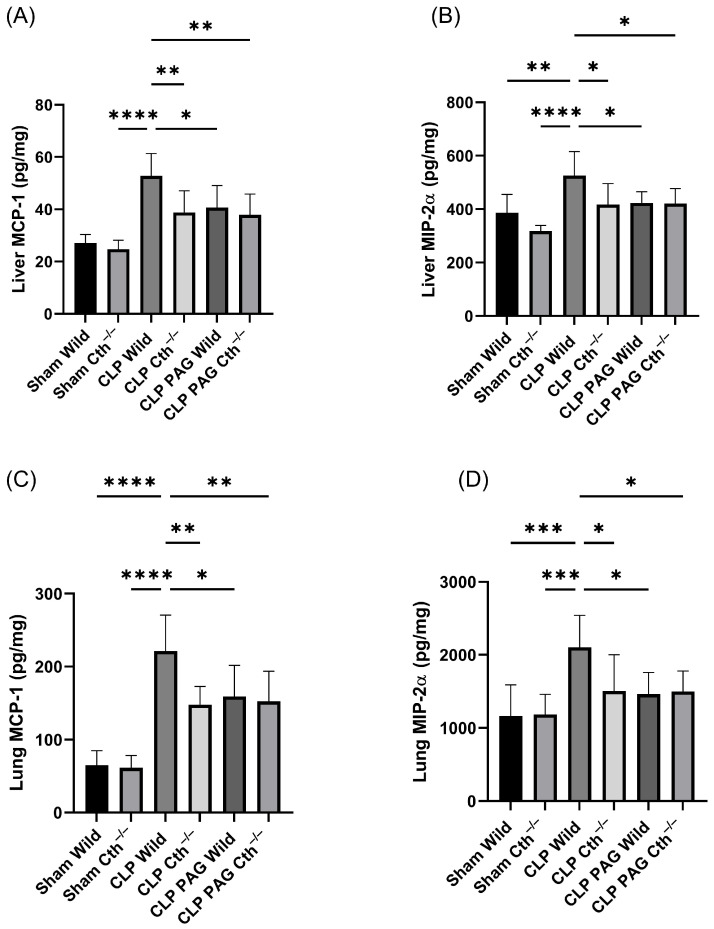
Impact of Cth inhibition or deletion on expression of liver MCP-1 (**A**) and MIP-2α (**B**) and lung MCP-1 (**C**) and MIP-2α (**D**) after 8 h of CLP-induced sepsis. CLP-induced sepsis in WT mice significantly elevated the pro-inflammatory chemokines MCP-1 and MIP-2α in the liver and lung more than in sham control. Cth^−/−^, PAG-treated WT, and PAG-treated Cth^−/−^ mice were demonstrated to reduce pro-inflammatory MCP-1 and MIP-2α expression in the liver and lung compared to WT CLP-induced septic mice. Results are expressed in pg/mg of protein. All data are represented as mean ± SD (*n* = 8). One-way ANOVA with post hoc Tukey test was employed. Statistical significance was determined at * *p* < 0.05; ** *p* < 0.01; *** *p* < 0.001; and **** *p* < 0.0001.

The concentrations of pro-inflammatory chemokines (MCP-1 and MIP-2α) were significantly elevated in WT CLP mice as compared to WT sham mice (Figure 4A–D). PAG-treated and Cth^−/−^ mice with CLP-induced sepsis demonstrated lower concentrations of pro-inflammatory chemokines in liver and lung homogenates than WT septic mice.

The concentrations of pro-inflammatory chemokines in liver and lung samples from PAG-treated Cth^−/−^ mice were not significantly different from the Cth^−/−^ group.

### 2.5. Effect of PAG Treatment and Cth^−/−^ on Adhesion Molecule Synthesis in Liver and Lung Following Sepsis

**Figure 5:**
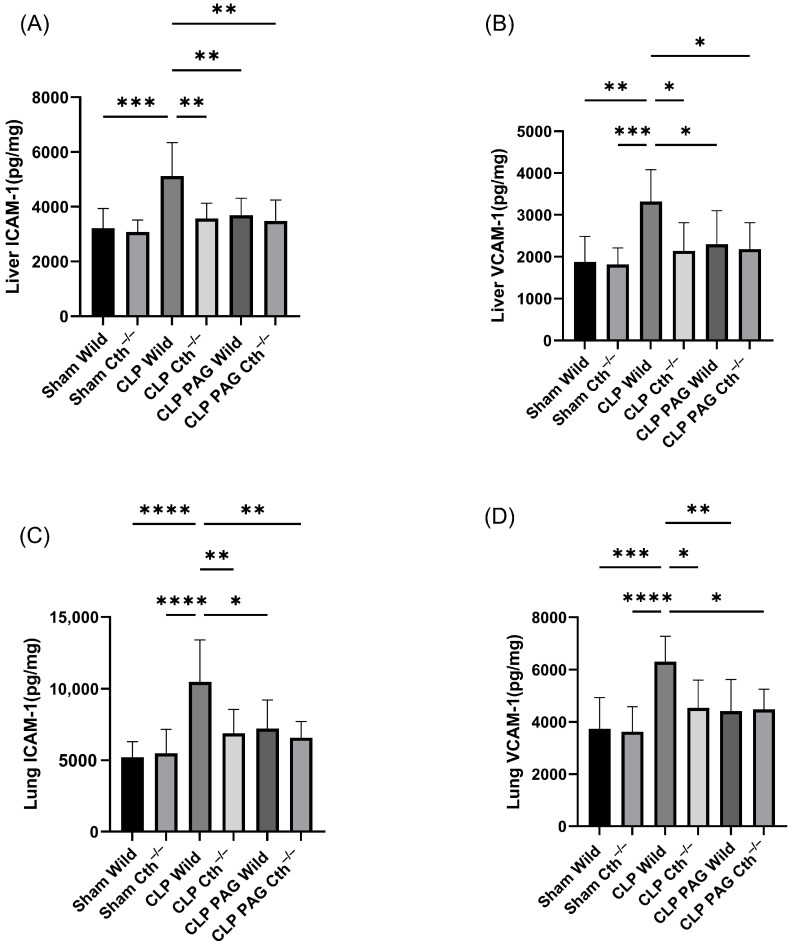
Impact of Cth inhibition or deletion on expression of liver ICAM-1 (**A**)/VCAM-1 (**B**) and lung ICAM-1 (**C**)/VCAM-1 (**D**) after 8 h of CLP-induced sepsis. WT CLP-induced septic mice significantly elevated pro-inflammatory adhesion molecules ICAM-1 and VCAM-1 in the liver and lung compared to sham operation controls. Cth^−/−^, PAG-treated WT, and Cth^−/−^ mice were shown to reduce pro-inflammatory ICAM-1 and VCAM-1 expression in the liver and lung compared to WT CLP-induced septic mice. Results are expressed in pg/mg of protein. All data are represented as mean ± SD (*n* = 8). One-way ANOVA with post hoc Tukey test was employed. Statistical significance was determined at * *p* < 0.05; ** *p* < 0.01; *** *p* < 0.001; and **** *p* < 0.0001.

The concentrations of pro-inflammatory adhesion molecules (ICAM-1 and VCAM-1) in liver and lung tissue were significantly elevated in WT CLP mice compared to WT sham-operated mice (Figure 5A–D). PAG-treated and Cth^−/−^ mice with CLP-induced sepsis demonstrated lower protein concentrations of pro-inflammatory adhesion molecules in liver and lung homogenate than WT septic mice. The concentrations of pro-inflammatory adhesion molecules in liver and lung samples from PAG-treated Cth^−/−^ mice were not significantly different from the Cth^−/−^ group.

### 2.6. Effect of PAG Treatment and Cth^−/−^ on Immunoreactivity of Liver ICAM-1 and VCAM-1 Co-Localised with Liver Sinusoidal Endothelial Cells

**Figure 6:**
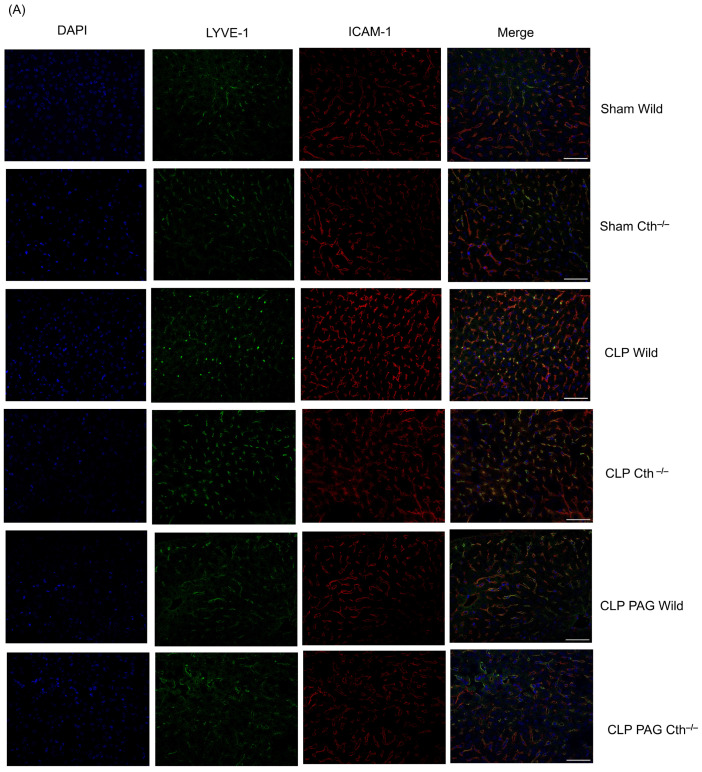

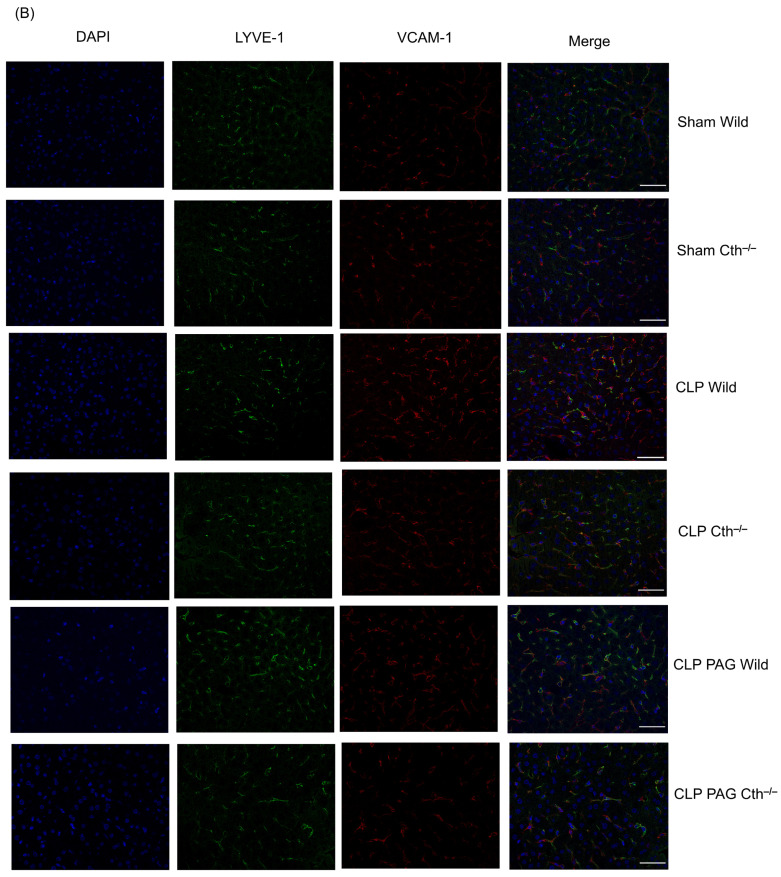

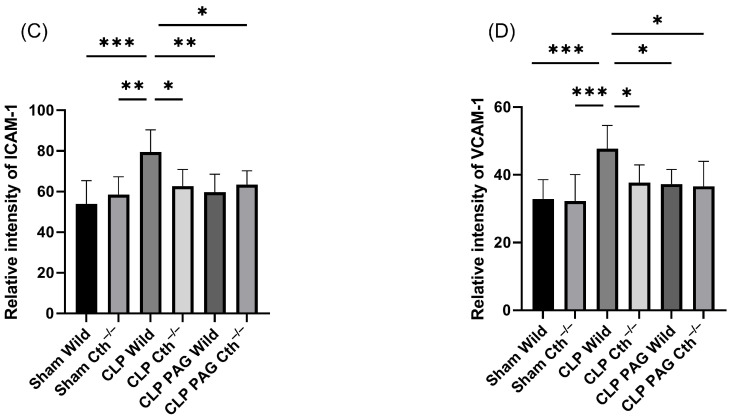
Representative images of ICAM-1 (**A**) and VCAM-1 (**B**) immunoreactivity (Cy3–red) co-localized with LYVE-1 (Alexa Fluor 488–green) after 8 h of CLP or sham operation. Scare bar: 50 μm. Semi-quantitative analysis of ICAM-1 (**C**) and VCAM-1 (**D**) immunoreactivity on LSEC were increased in CLP-induced WT mice compared to sham-operated control mice. Cth^−/−^ and PAG treatment on the WT and Cth^−/−^ group demonstrated decreased levels of ICAM-1 and VCAM-1 expression on LSECs compared to CLP-induced WT mice. All data are represented as mean ± SD (*n* = 8). One-way ANOVA with post hoc Tukey test was employed. Statistical significance was determined at * *p* < 0.05; ** *p* < 0.01; *** *p* < 0.001.

To quantify the localized ICAM-1 and VCAM-1 immunoreactivity within the liver, co-localization was performed with LYVE-1 to identify liver sinusoidal endothelial cells. Immunofluorescence microscopy showed increased immunoreactivity of ICAM-1 (Figure 6A,C) and VCAM-1(Figure 6B,D) co-localized with LYVE-1 in WT CLP-induced septic mice compared with sham-operated control mice. Cth^−/−^ and PAG treatment on WT and Cth^−/−^ attenuated the expression of ICAM -1 and VCAM-1 co-localized with LYVE-1 compared to WT CLP mice.

### 2.7. Effect of PAG Treatment and Cth^−/−^ on Immunoreactivity of Lung ICAM-1 and VCAM-1 Co-Localised with Pulmonary Endothelial Cells

**Figure 7:**
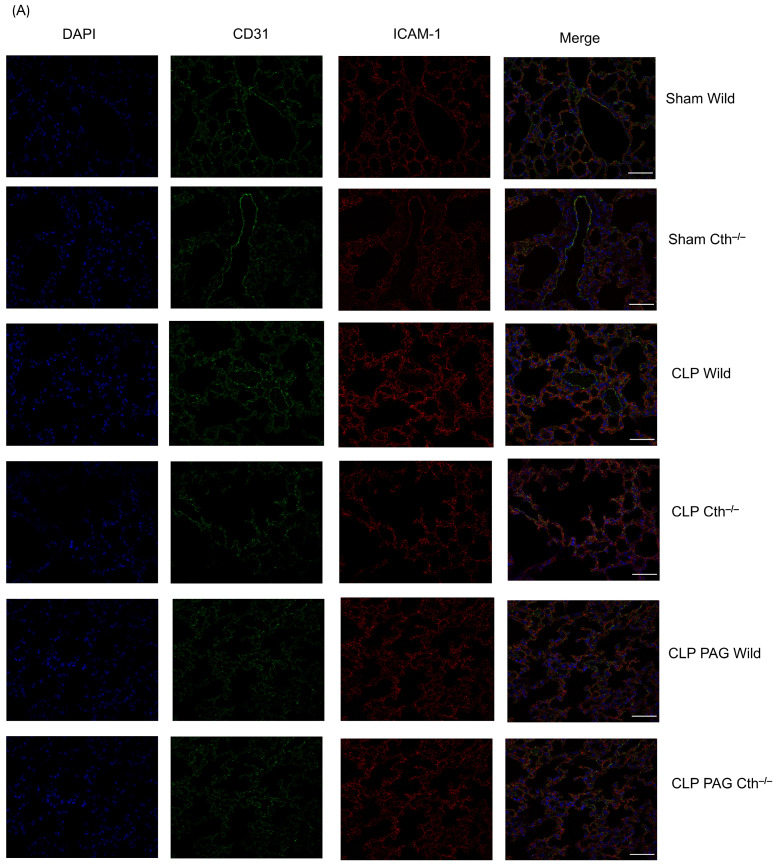

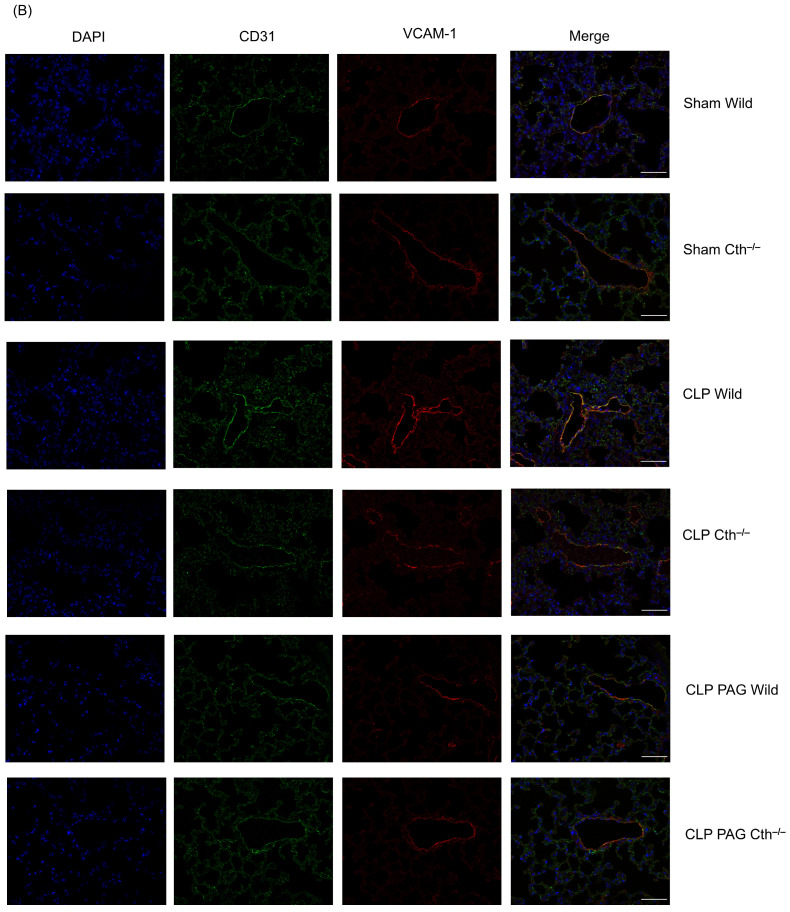

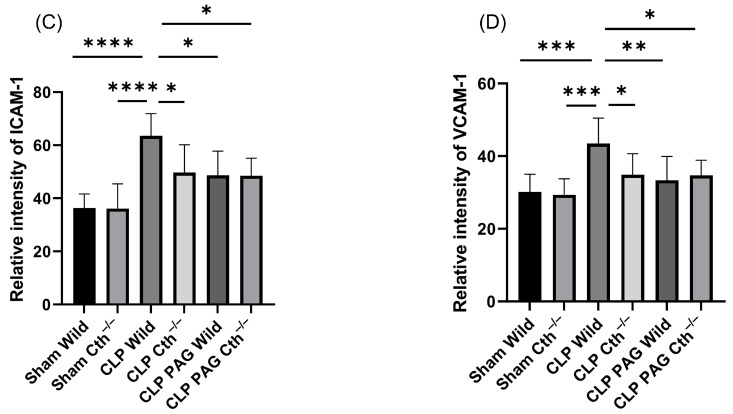
Representative images of ICAM-1 (**A**) and VCAM-1 (**B**) immunoreactivity (Cy3–red) co-localized with CD-31 (Alexa Fluor 488–green) after 8 h of CLP or sham operation. Scare bar: 50 μm. ICAM-1 (**C**) and VCAM-1 (**D**) immunoreactivity on pulmonary endothelial cells was significantly higher in WT CLP-induced septic mice than in WT sham-operated control mice. Cth^−/−^ and PAG treatment on the WT and Cth^−/−^ group showed lower levels of ICAM-1 and VCAM-1 immunoreactivity on pulmonary endothelial cells compared to CLP-induced WT mice. All data are represented as mean ± SD (*n* = 8). One-way ANOVA with post hoc Tukey test was employed. Statistical significance was determined at * *p* < 0.05; ** *p* < 0.01; *** *p* < 0.001; and **** *p* < 0.0001.

CD31 was used to identify the pulmonary endothelial cells, and the co-localized immunoreactivity of ICAM-1 and VCAM-1 with CD 31 was quantified. Immunofluorescence microscopy showed increased immunoreactivity of ICAM-1 (Figure 7A,C) and VCAM-1 (Figure 7B,D) co-localized with CD31 in WT CLP-induced septic mice than in sham-operated control mice. Cth^−/−^ and PAG treatment on WT and Cth^−/−^ attenuated the immunoreactivity of ICAM-1 and VCAM-1 co-localized with CD31 compared to WT CLP mice.

## 3. Discussion

Several studies have used different preclinical animal models to investigate the role of H_2_S in sepsis. Both the CLP-induced sepsis and LPS-induced endotoxemia models resulted in increased synthesis of H_2_S concentrations. PAG is the best-studied inhibitor of H_2_S synthesis derived from Cth. Studies using PAG have pointed to Cth as a key enzyme responsible for elevating circulating and tissue H_2_S, as well as tissue H_2_S-synthesizing activity. This showed a systemic inflammatory response and multiple organ damage in sepsis, suggesting a pro-inflammatory role of H_2_S in sepsis. In addition to Cth inhibition, PAG also inhibits other enzymes that use pyridoxal-5-phosphate as a co-factor [33]. Our recent study used Cth^−/−^ mice to specifically target the Cth-H_2_S pathway, and confirmed the key role of H_2_S synthesized by Cth in sepsis [4]. The pleiotropic effect of other genes, however, could potentially compensate for the loss of Cth activity in the KO model, but this has not been shown previously. The present study therefore employs two complementary and independent means of H_2_S inhibition in parallel to explore this over 8 h after CLP in a mouse model of polymicrobial sepsis. Furthermore, the effect of PAG treatment on Cth^−/−^ mice will shed light on whether PAG primarily exerts its therapeutic action through Cth or if other mechanisms are also at play. We have selected an 8 h time point of CLP-induced sepsis in this study as there is a significant elevation of chemokines and cytokines at this time point, which has been shown to correlate with the severity of sepsis [19]. In addition, Cth activity in the liver is found to peak at 4–8 h after CLP. Post 8 h, there is a slow decline in the liver Cth activity and plasma H_2_S concentration, leading to a restoration of levels similar to those observed in normal/sham-operated mice [19].

In this study, we found increased Cth expression and H_2_S-synthesizing activity in the liver and lung following CLP-induced sepsis. This increase correlated with elevated MPO activity and histological alterations in the liver and lungs. Interestingly, Cth^−/−^ and PAG-treated WT and Cth^−/−^ mice demonstrated a similar reduction in liver H_2_S-synthesizing activity and MPO activity in both organs, which reduced injury in these organs. Notably, PAG treatment in CLP-induced sepsis WT mice did not affect Cth expression but inhibited its activity in synthesizing H_2_S. These findings suggest that PAG does not affect Cth expression but rather inhibits the H_2_S-synthesizing activity of the enzyme.

In response to sepsis, the immune system triggers increased production of various pro-inflammatory mediators (such as cytokines, chemokines, and adhesion molecules) through transcriptional activation, such as NF-κB [34,35], which is activated by ERK1/2 phosphorylation [36,37,38]. Our results confirm previous studies using PAG and NaHS that have demonstrated that an acute increase in H_2_S concentrations is a key stimulus for the elevation of ERK1/2 phosphorylation and IκB deterioration, which subsequently induces the nuclear translocation of NF-κB in sepsis [19]. Recently, Cth deletion has been shown to attenuate liver and lung injury and the systemic inflammatory response by lowering ERK1/2 phosphorylation and the activation of NF-κB p65, as well as reducing the production of cytokines (TNF-α, IL-6, and IL-1β) and chemokines (MCP-1 and MIP-2α) [4]. These results suggest that the ERK1/2–NF-κB p65 signaling pathway may play a significant role in Cth/H_2_S-mediated inflammation during sepsis.

This study demonstrated that Cth^−/−^ CLP mice showed lower expression of ICAM-1 and VCAM-1 adhesion molecules compared to WT CLP mice in the liver and lung. Additionally, we found that PAG treatment in Cth^−/−^ mice had no effect on the expression of ERK 1/2 phosphorylation, NF-κB p65, or the production of chemokines (MCP-1 and MIP-2α) and adhesion molecules (ICAM-1 and VCAM-1) compared to Cth^−/−^ mice in liver and lung. However, a recent study has shown that PAG treatment in infected WT macrophages significantly reduced Mycobacterium tuberculosis CFU (colony-forming unit) counts even lower than in infected Cth^−/−^ macrophages [39]. Differences in dosages, treatment conditions, and models may contribute to these discrepancies.

ICAM-1 and VCAM-1 are adhesion molecules primarily expressed on endothelial cells that respond to cytokines in a time- and dose-dependent manner. The transcription of these adhesion molecules is dependent on transcription factors such as NF-κB and AP-1, which play an important role in the activation of endothelial cells and the elevated expression of adhesion molecules in inflamed microvessels in response to various stimuli [40,41,42]. Our study showed increased ICAM-1 and VCAM-1 immunoreactivity in co-localization with LYVE-1 in a sepsis model. Similarly, ICAM-1 and VCAM-1 immunoreactivity in CLP-induced mice increased in co-localization with CD31. This suggested there was increased expression of ICAM-1 and VCAM-1 predominantly on liver sinusoidal endothelial cells and pulmonary endothelial cells, respectively, during sepsis. This increase could be from the elevated expressions of cytokines and the NF-κB transcription factor observed. Organ injury during inflammation is mainly induced by the selective infiltration of leukocytes, which is regulated by the expression of adhesion molecules and chemokines in the vascular endothelium [43,44,45]. Pharmacological inhibition of Cth and gene deletion both resulted in reduced immunoreactivity of ICAM-1 and VCAM-1 in co-localization with LYVE-1 and CD 31. This demonstrated that Cth is responsible in the regulation of adhesion molecules in both liver sinusoidal endothelial cells and pulmonary endothelial cells. In animal models and patients, liver injury is associated with the capillarization/defenestration (reduced porosity, frequency, and diameter) of LSECs [46]. Cth^−/−^ mice demonstrated less defenestration and gap formation in LSECs following CLP-induced sepsis [4]. This indicates that Cth/H_2_S synthesis regulates adhesion molecules in endothelial cells, which may be responsible for the alteration in fenestration and gap formation in LSECs, resulting in liver injury during sepsis. Similarly, the reduction in adhesion molecule expression in pulmonary endothelial cells following sepsis through Cth inhibition (both pharmacological and genetic) may be responsible for the attenuation of lung injury in sepsis, as reduced levels of adhesion molecules in endothelial cells decrease leukocyte infiltration to the site of infection, resulting in reduced injury. The histological evidence from the liver and lung further substantiates these results. Cth inhibition (both pharmacological and genetic) protected the liver from ballooning degeneration and focal hepatocyte necrosis in sepsis. Similarly, there was a decrease in neutrophil infiltration, alveolar wall thickening, and fibrin deposition in Cth inhibition (both pharmacological and genetic) septic mice groups.

Employing two parallel complementary approaches has strengthened the evidence of H_2_S involvement in sepsis. This study also validated the use of PAG as a proper tool in Cth/H_2_S signaling in sepsis (inflammation), as no additional effect of PAG was observed in Cth^−/−^ mice. Furthermore, this is the first study to demonstrate the Cth/H_2_S signaling regulation of adhesion molecules in LSECs and pulmonary endothelial cells following sepsis. However, further investigation is needed to understand the precise mechanism of involvement of Cth/H_2_S-synthesis-mediated changes in the expression of adhesion molecules in the LSECs and pulmonary endothelial cells.

This study and other studies have demonstrated the role of H_2_S as a pro-inflammatory mediator in inflammation [5,7,13,47,48]. However, H_2_S has also been suggested as having an anti-inflammatory action, pointing to contradictory outcomes in various inflammatory diseases [49,50,51,52,53,54,55]. H_2_S may appear to have different pathogenic outcomes in different animal models but differences in the dosage, source of drug, and route and time of administration could also produce differing outcomes in the same model. 

**Figure 8:**
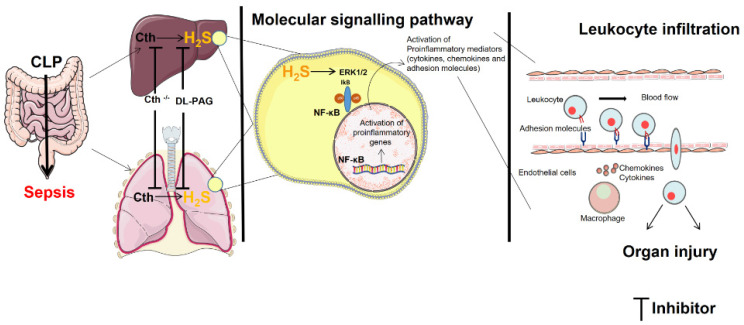
Hydrogen sulfide regulates liver and lung injury in CLP-induced sepsis. An increase in the expression of Cth results in the elevation in hydrogen sulfide synthesizing activity. This leads to the phosphorylation of ERK1/2, resulting in translocation and activation of NF-κB, thereby increasing the adhesion molecules’ expression on liver sinusoidal endothelial cells and pulmonary endothelial cells that results in liver and lung injury. The figure incorporates images sourced from Servier Medical Art, which is licensed under a Creative Commons Attribution 3.0 Unported License (https://creativecommons.org/licenses/by/3.0/).

In conclusion, this study has demonstrated a key role of adhesion molecules expressed on endothelial cells in the pro-inflammatory action of H_2_S in sepsis. PAG treatment in Cth^−/−^ mice showed no additional effect in the activation of ERK1/2 and NF-κB p65 signaling, chemokines, and adhesion molecules, attenuating tissue damage in liver and lungs compared to Cth deletion mice during CLP-induced sepsis. The PAG-treated WT mice also showed similar results. Cth^−/−^ mice and Cth inhibition using a pharmacological drug in both WT and KO mice demonstrated a decrease in the expression of adhesion molecules in LSECs and pulmonary endothelial cells (Figure 8). These results have important implications for our understanding of the mechanism by which H_2_S contributes to inflammation in sepsis, and could contribute to the development of novel therapeutic approaches for sepsis.

## 4. Materials and Methods

### 4.1. Induction of Polymicrobial Sepsis in Mice

All experimental procedures were authorized by the University of Otago Animal Welfare Office and Ethics Committee and conducted under the established guidelines. WT, Cth^−/−^ [56], and PAG-treated WT and Cth^−/−^ mice (C57BL/6J males, 25–30 g) were allocated to control or experimental groups in a randomized manner. WT mice were acquired from the Christchurch Animal Research Area, and the Cth^−/−^ mice were bred by mating Cth heterozygous mice as previously described. Forty-eight mice were used in total, divided into WT saline sham, Cth^−/−^ saline sham, WT saline sepsis, Cth^−/−^ saline sepsis, PAG-treated Cth^−/−^ sepsis, and PAG-treated WT sepsis (*n* = 8 each).

CLP-induced sepsis was performed with a few minor modifications to a previously described protocol [4]. Briefly, mice were mildly anesthetized using inhaled isoflurane (2%, 1 L/min O_2_). Sterile surgical procedures were employed during the CLP operation. PAG (50 mg/kg) was dissolved in saline and intraperitoneally (i.p) administered 30 min before CLP (CLP PAG WT and CLP PAG Cth^−/−^). Sham WT, sham Cth^−/−^, CLP WT, and CLP Cth^−/−^ were administered with saline intraperitoneally as a vehicle control. Disinfectant was applied after the removal of abdominal fur. A small midline incision through the skin, abdominal wall, and peritoneum was made to expose the caecum, which was ligated with 5.0 Silkam thread 8–10 mm from the end of the cecum without obstructing the bowel passage. Thereafter, a 22 gauge (22G)-sized needle was used to perforate the distal end of the cecum in two different locations. A small amount of feces was squeezed out through each hole; then, the bowel was repositioned. The abdomen was sutured with sterile permilene 5.0 thread. The same surgical procedure was performed on sham mice but without bowel perforation. Buprenorphine (Temgesic, 0.2 mg/kg) was administered by the subcutaneous route 45 min prior to and 3 h after the surgical procedure (sham or CLP) for analgesia. Eight hours after surgery, an IP injection of pentobarbital sodium (150 mg/kg) was administered to euthanize mice. Blood samples were taken from the right ventricle using heparinized syringes. Plasma was collected after centrifugation (1000× *g*, 5 min, at 4 °C). Sections of the liver and lung tissues were fixed in 10% formalin for more than 24 h, processed via a series of graded ethanol, and infused with paraffin wax. The rest of the tissue samples were kept at –80 °C for quantification of chemokines and adhesion molecules, H_2_S-synthesizing activity, and tissue MPO. 

### 4.2. Myeloperoxidase (MPO) Activity

Leukocyte sequestration into the liver and lung was quantified by measuring tissue MPO activity. Tissue samples were thawed, homogenized in 20 mM phosphate buffer (pH 7.4), centrifuged (13,000× *g*, 10 min, 4 °C), and the resulting pellet was resuspended in 50 mM phosphate buffer (pH 6.0) containing 0.5% *w*/*v* hexadecyltrimethylammonium bromide (Sigma-Aldrich, Dallas, TX, USA). The suspension was subjected to three cycles of freezing and thawing followed by sonication for 40 s. The reagent mixture consisted of supernatants (50 mL), tetramethylbenzidine (1.6 mM), sodium phosphate buffer (80 mM, pH 5.4), and hydrogen peroxide (0.3 mM) and this reagent was incubated at 37 °C for 110 s. The reaction was stopped with 50 μL of 2 M H_2_SO_4_, and absorbance was measured at 450 nm (Thermo Fisher Multiskan GO, Ratastie, Finland) and corrected for the protein content of the tissue sample using results from the Bradford protein assay [57]. The results are presented as a fold increase over a control group. 

### 4.3. H_2_S-Synthesizing Activity Assay

H_2_S-synthesizing activity in liver tissue was measured, according to the protocol described previously with minor modifications [4]. The liver tissue was homogenized in 20mM mM sodium phosphate buffer (pH 7.4) while kept on ice. The reaction mixture is composed of sodium phosphate buffer (20 mM, pH 7.4), pyridoxyal 5′-phosphate (10 μL, 18 mM), and l-cysteine (10 μL, 250 mM) in 230 µL of tissue homogenate. The reaction was performed in a tightly parafilm-sealed microfuge tube and initiated by transferring the tubes from ice to a water bath at 37 °C. An amount of 250 µL of zinc acetate (1% *w*/*v*) was added after 30 min of incubation in order to capture generated H_2_S. Then, a mixture of *N*, *N*-dimethyl-*p*-phenylenediamine sulfate (20 mM) in 7.2 M HCl and FeCl_3_ (30 mM) in 1.2 M HCl (133 μL, in 1:1 ratio) was added. Subsequently, the samples were incubated in the dark at room temperature for 20 min. After that, 10% (*v*/*v*) trichloroacetic acid was added to denature the protein and end the reaction. The supernatant was collected after the centrifugation and absorbance was measured with a 96-well microplate spectrophotometer at 670 nm. The H_2_S concentration was measured against a calibration curve of Na_2_S. The results are expressed as nmole H_2_S/mg protein after being normalized for the tissue sample’s protein concentrations as determined via the Bradford assay.

### 4.4. Western Blotting

Liver and lung tissue was lysed with homogenizer (Labserv, Fisher Scientific, Singapore) in lysis buffer (ice-cold RIPA buffer and protease inhibitor). Then, homogenates were centrifuged at 10,000× g for 10 min at 4 °C. Proteins (30 µg) were separated using 10% sodium dodecyl sulfate-polyacrylamide gel via electrophoresis and transferred onto a polyvinylidene difluoride (PVDF, Bio-Rad, Hercules, CA, USA) membrane via wet transfer for 1 h. After blocking the membrane with 0.1% (*w*/*v*) Tween 20 in tris-buffered saline (TBST) containing 5% (*w*/*v*) non-fat dry milk at room temperature for 1 h, the membrane was incubated overnight with primary antibody (Table 1) at 4 °C. The membrane was washed with TBST, incubated for 1.5 h with secondary antibody (Table 1) at room temperature, and detected with chemiluminescent reagent (Supersignal West Pico, Thermo Scientific, Waltham, MA, USA). The detection was carried out using a chemi-doc instrument (Uvitec, Cambridge, UK).

### 4.5. Double-Immunofluorescence Staining of Paraffin Sections

Liver and lungs were extracted and fixed in 4% buffered paraformaldehyde. The fixed tissue was processed and embedded in paraffin. The paraffin-embedded tissues were sectioned to 4 μm thickness using a LEICA microtome (HistoCore BIOCUT R, Leica Biosystems, Mt Waverley, Australia) and attached to charged glass slide. Sections were deparaffined and hydrated followed by antigen retrieval by cooking slides in Tris EDTA pH 9.0 buffer at 100 °C for 4 min in a pressure cooker. The slides were left to cool in the buffer for 2 h. The slides were rinsed in water 3 times, followed by washing in PBS for 5 min. The tissue sections were permeabilized in 0.1% (*w*/*v*) Tween 20 in phosphate-buffered saline (PBST) buffer for 15 min at 4 °C. This was followed by washing sections with PBS 3 times for 5 min. The slides were drained properly without letting the section dry out. An ImmEdge pen (H-4000, Newark, CA, USA)was used to draw a hydrophobic barrier around the tissue section. Sections were blocked with 10% normal donkey serum at room temperature (humidifying chamber) for 1 h. After this, the sections were incubated in primary antibody (1:100 for LYVE-1, 1:1000 for ICAM-1, 1:200 VCAM-1, and 1:500 for CD31) diluted in 1% normal donkey serum overnight at 4 °C (in humidifying chamber) (Table 1). Slides were washed 3 times with PBS. This was followed by secondary antibody (Table 1) incubation for 2 h with DAPI (4′,6-diamidino-2-phenylindole) at room temperature. Slides were washed 3 times with PBS. Sections were mounted with anti-fading solution and observed with an epifluorescence microscope (Carl Zeiss, Oberkochen, Germany). 

Mean fluorescence intensity (MFI) was measured using an automated region of interest selection based on a signal threshold using Fiji ImageJ (downloaded at https://fiji.sc/). This measurement procedure was followed according to a protocol described previously with minor modifications [58]. In the previous protocol, the interested stain (targeted protein) was selected using an automated region of interest based on a signal threshold and then mean intensity was directly measured. In this modified protocol, the selection of LYVE-1 or CD31 (Alexa Fluor 488–green) staining was performed with an automated region of interest based on a signal threshold. The mean intensity of staining of targeted proteins (Cy3–red), which was measured from the automated region of interest (e.g., yellow ROI’s) selection obtained above from LYVE-1 or CD 31 (colonized of targeted protein in endothelial cells-LYVE-1). The background intensity for each image was calculated as the mean intensity within the blue regions of interest (ROIs). The intensity of layer-specific staining (ICAM-1 and VCAM-1) proteins was determined by subtracting the mean target protein staining intensity from the background staining intensity. This calculation was performed using 8 confocal images for each group and target protein (ICAM-1 and VCAM-1).

### 4.6. Histological Analysis

The liver and lung samples were extracted and fixed in 4% buffered paraformaldehyde. The fixed tissue was processed and embedded in paraffin. The samples were sectioned (4 µm), stained with hematoxylin/eosin (H&E), and examined via light microscopy using a Leica microscope. Organ (liver and lung) injury was scored double-blinded, involving an experienced pathologist (A.M.) as one of the individuals responsible for the assessment with regard to different treatment groups. Liver sections were assessed for liver injury (ballooning degeneration and focal hepatocyte necrosis) and scored for liver injury (ballooning degeneration) in 2 random fields at 10× magnification (*n* = 8 per condition) as previously described [59]. Lung pathology was assessed using a scoring system adapted from the American Thoracic Society guidelines [60]. The lung injury was based on the extent of fibrin deposition, leukocyte (neutrophil) infiltration, and alveolar wall thickening. For scoring, 3 random fields were counted per slide (*n* = 8 per condition) at 40× magnification.

### 4.7. NF-κB Activity Assay

NF-κB activation was measured in the nuclear extract of liver and lung tissues as per the manufacturer’s instructions using a TransAM NF-κB p65 transcription factor assay kit (Active Motif, Wangarra, Australia). Nuclear extracts (20 µg) were incubated in a 96-well plate with complete lysis buffer for 1 h before being incubated with a specific primary antibody against NF-κB p65 for 1 h. After, a secondary antibody conjugated to horse radish peroxidase (HRP) was added for detection. The microplate reader measured the absorbance of the enzymatic product at 450 nm. The wild-type or mutated consensus oligonucleotides were incubated in the wells to determine the assay’s specificity. The results are presented as a fold increase over a control group.

### 4.8. Enzyme-Linked Immunosorbent Assay

The protein concentrations of chemokines (MCP-1 and MIP-2α) and adhesion molecules (ICAM-1 and VCAM-1) were measured in liver and lung with a sandwich EILSA using DuoSet ELISA kits by R&D System (Minneapolis, MN, USA) as directed in the manufacturer’s protocol. Liver and lung homogenates were prepared by homogenizing 20 mg of tissue in 1 mL of sodium phosphate buffer (20 mM, pH 7.4). The homogenates were centrifuged at 15,000× *g* for 15 min at 4 °C. The supernatant was used to measure adhesion molecule and chemokine concentrations. Measurements were corrected for the tissue sample’s protein levels using Bradford protein assay and expressed as picogram per milligram of protein. 

### 4.9. Statistical Analysis

The data are reported as mean ± SD and assessed for Gaussian or normal distribution using the Shapiro–Wilk test. For normally distributed data, one-way ANOVA with post hoc Tukey’s test was performed. If the data were not normally distributed, the non-parametric Kruskal–Wallis test was used. Statistical analysis in this study was conducted using GraphPad Prism software (version 9, San Diego, CA, USA). Statistical significance was set at *p* < 0.05.

## Figures and Tables

**Table 1:** Primary and secondary antibodies used in this study for Western blotting and immunofluorescence microscopy.

Product	Antibody/Type	Source Catalogue No.	Dilution
**Western blotting**			
CTH	Primary/Mouse monoclonal	Abnova, Taipei City, Taiwan/H00001491-M01	1:1000
GAPDH	Primary/Rabbit polyclonal	Santa Curz, Dallas, TX, USA/sc-25778	1:2000
ERK1/2	Primary/Rabbit monoclonal	Cell Signaling, Danvers, MA, USA/137F5	1:2000
p-ERK1/2	Primary/Rabbit monoclonal	Cell Signaling, Danvers, MA, USA/93H1	1:2000
Goat anti-mouse HRP	Secondary	Santa Curz, Dallas, TX, USA/sc-2005	1:20,000
Goat anti-rabbit HRP	Secondary	Abcam, Cambridge, UK/ad6721	1:20,000
**Immunofluorescence**			
ICAM-1	Primary/Goat polyclonal	R&D System, Minneapolis, MN, USA/AF796	1: 1000
VCAM-1	Primary/Goat polyclonal	R&D System, Minneapolis, MN, USA/AF643	1: 200
LYVE-1	Primary/Rabbit polyclonal	Abcam, Cambridge, UK/ab14917	1:100
CD31	Primary/Rabbit polyclonal	Abcam, Cambridge, UK/ab124432	1:500
Donkey anti-goat	Secondary/Texas Red	Abcam, Cambridge, UK/ab6883	1:1000
Donkey anti-rabbit	Secondary/FITC	Abcam, Cambridge, UK/ab6798	1:1000

## Data Availability

All data generated or analysed during this study are included in this published article.
